# Novel hazards of waterpipe tobacco and the benefits of stop smoking in men, a prospective cohort study

**DOI:** 10.1038/s41598-023-34388-1

**Published:** 2023-05-05

**Authors:** Hung Dinh Kieu, Can Van Phan, Hoc Hieu Tran, Ngoan Tran Le

**Affiliations:** 1 Dept. of Neurosurgery and Spine, Hanoi Medical University Hospital, Hanoi, Viet Nam; 2 Hanoi University of Public Health, Hanoi, Viet Nam; 3 Institute of Research and Development, Duy Tan University, Da Nang City, Viet Nam; 4 Department of Occupational Health, Hanoi Medical University, Hanoi, Viet Nam

**Keywords:** Cancer, Risk factors

## Abstract

Waterpipe smoking is an emerging epidemic and a severe public health problem worldwide. Observational studies on the hazards of a specific new waterpipe tobacco product are timely needed. The objectives were to analyze how dangerous waterpipe tobacco smoking is on the causes of all mortality, including cancer, and how effective smoking cessation is for improving health. We analyzed the hazards of exclusive waterpipe smoking through a prospective cohort study in Northern Vietnam. We obtained exposure data on the smoking status of specific cigarette and waterpipe and smoking cessation histories from each study participant. The outcome includes deaths due to all causes. The cause of death for each case is determined based on medical records. HR (95%CI) was estimated using a Cox proportional-hazards–regression analysis for overall mortality and all cancers. The ever-cigarette smoking group as the reference group, the exclusive waterpipe smoking group had a statistical increase in the risk for overall mortality HR (95% CI): 1.63 (1.32, 2.00), and all cancers HR (95%CI): 1.67 (1.18, 2.38). The risk of death increased statistically in the group of waterpipe smoking over 20 years for overall mortality HR (95%CI): 1.82 (1.45, 2.29), and all cancers HR (95%CI): 1.91 (1.27, 2.88). After stopping smoking, the risk of death decreased steadily. The risk of death was reduced by 41% for overall mortality HR (95%CI): 0.59 (0.39, 0.89), and 74% for death from cancers HR (95%CI): 0.26 (0.08, 0.83) after ten years or longer of cessation. Life expectancy was shortened by more than six years for the group of exclusive waterpipe smokers compared to non-smokers. This study found new novel hazards of exclusive waterpipe tobacco smoking. The findings are scientific evidence for developing strategies, policies, and budget allocations to control this novel tobacco product and promote cessation to improve life expectancy.

## Introduction

The most dangerous public health problem worldwide for centuries has been tobacco smoking. Tobacco smoke is the cause of death for over 8 million people each year. About half of smokers will get sick and die from tobacco-related diseases. Among tobacco smoking forms, the cigarette is the most common, followed by waterpipe, cigars, roll-your-own tobacco, pipe tobacco, bidis, and kreteks^1^. In the last two decades, the trend of smoking waterpipe in the United States has elevated among people aged 18 and over, with an average annual increase of 9.4% (7.8%-11.0%)^2^. Not only in the U.S., but waterpipe smoking is also an emerging epidemic with an increasing trend and a severe public health problem worldwide^3^.

**Figure 1:**
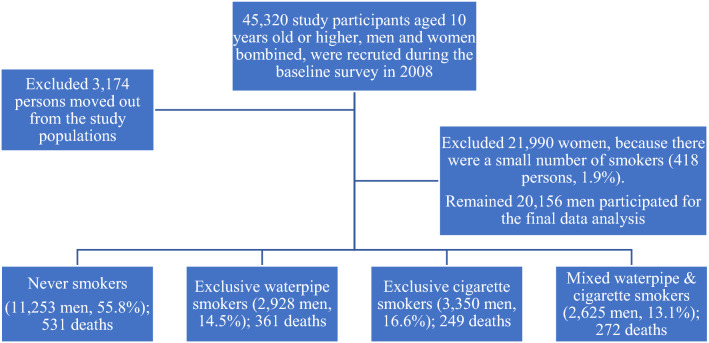
Eligible study participants aged ten years old or higher in men by smoking status during 2008 baseline survey and the number of cumulative deceased persons by the last followed up in 2019.

Waterpipe smoking has been reported in the last century in Viet Nam. During the 1990s, about one of the seventh smokers (14.9%) smoked waterpipe tobacco, most of which were found in the rural areas in the northern country^4^. There were 15.6 million people aged 15 + who currently smoked tobacco in 2015, and 29.8% of them smoked waterpipe tobacco^5^. The estimated prevalence of current waterpipe smokers increased from 13.0% to 13.7% in men and from 0.1% to 0.2% in women between 2010 and 2015, respectively^5^. The impact of this specific tobacco form on people’s health is neglected in the country. The baseline survey in 2008 of the present prospective cohort study has highlighted the proportion of men study participants of exclusive waterpipe smokers (14.5%), exclusive cigarette smokers (16.6%), and mixed waterpipe and cigarette smokers (13.1%), Fig. 1.

The results of a study in the U.S. have suggested that waterpipe smoking is as dangerous as cigarette smoking^6^. Another European research has observed that the risk of cancer caused by cigarette smoking was highest, followed by smoking waterpipes and cigars^7^. The level of deep inhalation of waterpipe smoke is the specific behavior of waterpipe smokers because a vacuum is created in the base to inhale smoke by the smokers^3^. Moderate or deep inhalation of waterpipe tobacco smoke significantly increases the risk of cancers^6,7^.

The unique feature of the waterpipe smoking in Vietnam is that the smoker uses the flame to burn it directly on the rounded and tight tobacco material, combined with maximum inhalation and depth^8,9^. In contrast, people use charcoal to produce smoke elsewhere to burn tobacco^3^. Compared to cigarettes, waterpipe smoke was found to have higher concentrations of nicotine, carbon monoxide, carbonylic compounds, heavy metals, tar, and polyaromatic hydrocarbons^3^. The objectives were to analyze how dangerous waterpipe smoking is on the causes of all mortality, including cancer, and how effective smoking cessation is for improving health.

## Methods

We analyzed the hazards of exclusive waterpipe smoking compared to exclusive cigarette smoking from 2008 to 2019 through a prospective cohort study of people aged ten years and older living in nine communities in Northern Vietnam. The Scientific Committees of the Ministry of Health and the Ministry of Science and Technology approved the research protocol before conducting a baseline survey for the first research grant awarded in 2006. The study protocol for the second awarded research grant was approved by the IRB-Hanoi Medical University, Vietnam (Approval number NCS33/HMU-IRB dated 29 March 2019) for ethics in biomedical research implementation.

### Research design

Under the support of the Ministry of Health and the Ministry of Science and Technology, the Hanoi Prospective Cohort Study was established in 2008 as part of the nationally awarded research project entitled “Molecular Epidemiology Study on Stomach and Colorectal Cancers in Viet Nam”. The Hanoi Medical University managed the research grant, and the Principal Investigators were the university faculties and established researchers.

Participating study populations were included based on reliable local facilities of the state commune health station in Hanoi city and the neighboring provinces of Hung Yen and Phu Tho. The inclusion criteria included the population size of each commune was less than 15,000 residents for further feasible following-up of the study participants; the population was stable to minimize migration and loss of study participants; available daily medical records of inpatient and outpatients who have visited the state commune health station for receiving health services, active of weekly updated mortality registration of the deceased persons who have been living in the commune and have registered, having an agreement in written documents of the local government authority of People Committee to conduct a baseline survey in 2008 and follow-up activities from this time onward. Five communes of Hanoi city, three communes of Hung Yen province, and one commune of Phu Tho province were included in the present prospective cohort study. At least one physician has been appointed to work full-time in each commune health station. This medical doctor will serve as a family doctor and oversee morbidity cases and the underlying causes of death for each deceased person. These outcomes of the state commune health station will be used as part of the outcome of the present prospective cohort study.

### Selection of household and study participants

Living in these selected nine communes, there were over 17,000 registered households. After getting the agreement in written documents of the local government authority of the People Committee to conduct a baseline survey in 2008, the officers of nine communes had to invite each family to participate in the study verbally. To avoid selection bias, we plan to complete a baseline survey for all registered households and family members aged ten years and older. After two weeks, over 12,000 households from these nine communes had an agreement and registered to participate in the study. The criteria of a study participant inclusion included the registered residents of each commune for the benefit of healthcare services, agreeing to participate in the study for many decades after completing a baseline survey, not suffering from severe chronic diseases including cancer, physical presence at their household while conducting the study, aged ten years old or higher. The exclusion criteria included temporary residents at the selected communes who did not agree to participate in the study, suffered from severe chronic diseases, including cancer, were absent from the household while conducting a baseline survey, and were under 10.

### Conducting a baseline survey

The interviewers were third-year medical students of Hanoi Medical University who had completed four days of training and practices to use the designed and validated demographic lifestyle and semi-quantitative food frequency questionnaires. 30–40 trained interviewers worked for seven consecutive days at each commune with the support of local village health volunteers and officers of the commune health stations. A household visit and a face-to-face interview were conducted to collect data on demographic and household facilities and individual study variables using the printed handout of the demographic lifestyle and semi-quantitative food frequency questionnaires. The cross-sectional survey was conducted to collect data from all registered households and study participants.

For the sample size, we designed the prospective cohort study^10^ to examine the individual cancer site after the first decade of the following-up of stomach and colorectal cancers. The results have been published elsewhere for the association between waterpipe tobacco smoking and stomach cancer^9^. Therefore, the present study of all causes will have a reliable, strong power to detect the hazards of waterpipe smoking and the benefits of stopping smoking in men.

The entrance survey was completed in 2008 for all 45,320 people aged ten and over for both sexes. From 2008 to 2019, 3,174 persons moved out from the study populations and could not contact them to determine the migration date. Therefore, they were excluded from the study populations. Since there were 21,990 women, but only 418 (1.9%) had ever smoked in their lifetime, this number and proportion were so small that it was excluded from further analyses. Among 20,144 eligible men, 11,253 (55.8%) never smoked, 2,928 (14.5%) only smoked exclusive waterpipe tobacco, 3,350 (16.6%) smoked exclusive cigarettes, 2,625 (13.1%) had mixed smoking these two products of waterpipe and cigarettes, Fig. 1.

### ***Designed demographic ***lifestyle*** and semi-quantitative food frequency questionnaires***

The exposure variables were waterpipe and cigarette tobacco smoking. The covariate variables of demographic lifestyle and diet of the estimated macronutrient intake were derived from the designed demographic lifestyle and semi-quantitative food frequency questionnaires. The lifetime smoker definition was the person who has completed at least one course of waterpipe tobacco smoking and one cigarette daily for 30 consecutive days. During a face-to-face interview managed by the interviewers at the household, each study participant confirmed their smoking status by themselves and the head of the family. The raw data of smoking persons was the average number of waterpipe and cigarettes smoked daily or weekly, the age at started smoking, and the number of years of smoking for the current smokers. For the smokers but currently not smoking, the collected data included the average number of waterpipe and cigarettes smoked daily or weekly, the age at started smoking, the number of years of smoking, and the number of years they stopped smoking. Variables of lifestyles other than smoking included drinking alcohol and other beverages.

Demographic and health history data included current height and weight, education, history of hypertension, diabetes, blood transfusion, hepatitis B virus vaccine, abdominal symptoms, family history of cancer, and prostate symptoms among men. Data on socioeconomics included available fridges at home and other household suppliers and facilities and working conditions of working persons. The diet data had food frequency intake of food groups of fruits, vegetables, fish, meats, rice, and other cereals, salt, sauces, and spices. The data on cooking methods included fermented foods, salty foods, fried fish, meats, eggs, barbecued meats, fish, others, grilled chicken and poultry, fish, and meat. The designed demographic lifestyle and semi-quantitative food frequency questionnaires were validated^11^ to be reliable regarding macronutrient intake and feasible data on smoking published elsewhere^8,9,12^.

### ***Assessment of ***smoking*** status***

From the collected raw data on smoking, the derived data included never-smokers, exclusive waterpipe tobacco smokers, exclusive cigarette smokers, and mixed smokers. Detailed data on smoking status had the average number of waterpipe and cigarette smoking daily, age at started smoking, the number of years smoking, and the number of years stopped smoking for each group of exclusive waterpipe tobacco smokers, exclusive cigarette smokers, and smokers of these two products of waterpipe and cigarettes, Fig. 1. The reason to count smoking people aged from 10 years old is to avoid underexposure assessment. Because during the 1990s, the median age at smoking initiation was 19.5 years^4^, we believed there might be under-registration of child and adolescent smokers aged under 15 years old in the study populations.

The study participants provided detailed information on smoking status during direct household interviews with the trained interviewers using the designed questionnaires. Information on smoking status was whether they had smoked in their lifetime or had never smoked for each type of cigarette and waterpipe tobacco. Current smokers give information on the years smoked and the average number of cigarettes smoked daily. Current nonsmokers who smoked in the past provided information on the years they smoked, the average number of cigarettes smoked daily, and the number of months they quit smoking. The study participants provided the information independently for each type of cigarette and waterpipe.

The variables obtained from the information for each tobacco type were compared for each individual to determine the age of smoking initiation (the earliest time to smoke a waterpipe and tobacco completely), the average number of cigarettes they smoked per day, the number of years they smoked; the number of years they quit smoking; the total number of cigarettes they smoked in a lifetime; the total number of years they successfully quit smoking (the latest time to stop smoking cigarette or waterpipe). Assessment of smoking status has been published elsewhere^6^.

### ***Follow-up for ***the*** outcome***

Each study participant and each household was in an ID code. The collected raw and derived data and the quarterly updated data were also managed using the ID. Data was handled by two IT experts and backup for security. A censored procedure for each study participant was performed during the following-up. A quarterly update of data was made by each commune health station about the survival status of the study participants. The start time of follow-up is the date of completing information for the questionnaire questions. The final follow-up time is the date of confirmation of their health status in 2019 as being alive in the participating study community. For people who have died or moved, the last day of study follow-up is the date of death or transfer. Person-year was calculated for each study participant.

We focused the study period from 2008 to 2019 for all participants on deaths due to all causes and cancers. Those who died or migrated were compared from three independent sources: the death list recorded in the population at the state commune health station, the death or migration list of the data of the health management unit of maternal and child health and family planning, and the death or migration list of the data from the Justice unit of the state commune people's committee. This comparison is for complete and non-duplicate identification of the list of deaths and migrants.

The cause of death for each case is determined based on medical documents from the medical examination and treatment records kept at the health facilities, which are the state commune health station, the district hospitals, the provincial hospitals, the central hospitals, or other hospitals where the patient was last examined and treated. The cause of death was coded according to ICD-10.

### Data analysis

The overall risk of death from all causes and cancers in the group of exclusive waterpipe smokers compared with exclusive cigarette smokers was analyzed. In the dose–response analysis for the exclusive waterpipe smokers, the years smoking was divided into three groups of less than 11 years, 11–20 years, and over 20 years. The reference group for this analysis was the group of exclusive cigarette smoking. To analyze the effect of reducing the risk of death after quitting waterpipe smoking, this study group was divided into quartile groups of less than four years (as a reference group), 4–7 years, 8–10 years, and over ten years. Kaplan–Meier survival estimates were performed for three groups of exclusive waterpipe smokers, both waterpipe and cigarette smokers, and exclusive cigarette smokers. Multivariable adjusted HR (95%CI) was estimated using a Cox proportional-hazards–regression analysis for all cancers, controlling for possible confounding factors of age groups (10–19, 20–29, 30–39, 40–49, 50–59, 60–69, 70–79, 80 +); Education level (< seven years, 7 + years, unknown); Available fridge at home (yes/no, unknown, as an indicator of social-economic status); BMI (kg/m2, < 18.5, 18.5 ≤ 23, 23 + , unknown); Alcohol consumption (yes/no, unknown); Age at started smoking; Cumulative number of smokes lifetime; Years of quit smoking; Family history of cancer; Total energy intake (Kcal/day, quintiles); Protein intake (g/day, quintiles); Lipid intake (g/day, quintiles); Carbohydrate intake (g/day, quintiles). For all causes of death, we have added the additional indicator of the health history of hypertension for adjustment. All *p*-values were two-sided, and *p* ≤ 0.05 (alpha value) was considered to indicate statistical significance.

### Ethics approval and consent to participate

The authors confirm to follow the study protocol that was approved by the Ethics Committee of IRB-Hanoi Medical University, Vietnam, for ethics in biomedical research implementation (Approval number NCS33/HMU-IRB dated 29 March 2019) and the IRB-International University of Health and Welfare, Japan (Approval number 21-Ig-92 dated on 21 August 2021). The study is performed without intervention, a secondary analysis using existing data. We used the method of anonymously. Data is saved into a USB and private computer hard disk with a password. The principal investigator secured USB and computer and will only allow other people to go through them if research team members. The data will be saved for ten years after publication.

All methods were performed following relevant ethical guidelines and Vietnam's national regulations. We obtained verbal informed consent from 12,212 households and their family members before conducting an interview using the designed baseline survey questionnaire in 2008. All answers about smoking habits, diet-related factors, and family history will be anonymous by numbers. According to the guideline for epidemiological study in Vietnam and Japan in 2002, written informed consent was not required for observational research based on a questionnaire survey. Completion and return of the questionnaire were considered implied consent.

## Results

Of the 3,073 people who smoked exclusive waterpipe tobacco, 87.9% were current smokers. Among ever-exclusive cigarette smokers, the proportion of current smokers was lower at 70.9% (2,492 of 3516 participants) than waterpipe smokers, T-test *p* = 0.000, Table 1. The mean number of tobacco smoking per day and age at started smoking in the exclusive waterpipe was higher than the exclusive cigarette (10.45 versus 8.58), T-test *p* = 0.000 and (25.28-year-old versus 24.36-year-old), T-test *p* = 0.000, respectively. Smoking duration (Years) was similar between the exclusive waterpipe and exclusive cigarette (15.23 years versus 15.28 years), with T-test *p* = 0.375. The prevalence of waterpipe smokers was close to that of cigarette smokers (14.5% vs. 16.6%) in this study population. In addition, the most waterpipe and cigarette mixed smoking was 13.1%, Fig. 1.

**Table 1:** The proportion of former and current smokers by types of tobacco smoking, men and women combined.

Types of tobacco smoking	Former	Current	Total
Exclusive waterpipe smoking			
Number of participants	371	2,702	3,073
Percent (%) &	12.07	87.93	100
Exclusive cigarette smoking			
Number of participants	1,024	2,492	3,516
Percent (%) &	29.12	70.88	100
Mixed smokers			
Number of participants	691	2,041	2,732
Percent (%) &	25.29	74.71	100

T-test *p* = 0.000.

Compared with non-smokers, the risk of death from all cancer was statistically significantly increased for exclusive cigarette smokers, exclusive waterpipe smokers, and both waterpipe and cigarette smokers, HR (95% CI): 1.89 (1.41, 2.54), HR (95%CI): 2.84 (2.17, 3.72), HR (95%CI): 2.18 (1.60, 2.97), respectively, Table 2.

**Table 2:** Risk of death due to all causes and cancers caused by tobacco smoking status in men.

			All causes			All cancers	
Smoking status	person-year	Cases	Multivariable adjusted HR (95%CI) #	*P*	Cases	Multivariable adjusted HR (95%CI) &	*P*
Never smoker	122,453	531	1.00		94	1.00	
Mixed waterpipe cigarette smoking	27,794	275	1.45 (1.25, 1.68)	0.000	73	2.18 (1.60, 2.97)	0.000
Exclusive waterpipe	30,957	361	1.36 (1.19, 1.55)	0.000	126	2.84 (2.17, 3.72)	0.000
Exclusive cigarette	36,327	249	0.96 (0.82, 1.11)	0.569	86	1.89 (1.41, 2.54)	0.000

Adjusted for age groups (10–19, 20–29, 30–39, 40–49, 50–59, 60–69, 70–79, 80 +); Education level (< 6 years, 7 + years, unknown); Available fridge at home (yes/no, unknown); BMI (kg/m^2^, < 18.5, 18.5- < 23, 23 + , unknown); Alcohol consumption (yes/no, unknown); Family history of cancer; Total energy intake (Kcal/day, quintiles); Protein intake (g/day, quintiles); Lipid intake (g/day, quintiles); Carbohydrate intake (g/day, quintiles). # adjusted for “&” and additional health history of hypertension.

When taking the ever-cigarette smoking group as the reference group, the exclusive waterpipe smoking group had a statistically significant increase in the risk for overall mortality HR (95% CI): 1.63 (1.32, 2.00), and all cancer HR (95%CI): 1.67 (1.18, 2.38), Table 3.

**Table 3:** Compared to exclusive cigarette smoking, the risk of death due to all causes and cancers among men waterpipe tobacco smokers.

			All causes			All cancers	
Smoking types	person-year	Cases	Multivariable adjusted HR (95%CI) #	*P*	Cases	Multivariable adjusted HR (95%CI) &	*P*
Exclusive cigarette smoking	36,227	249	1.00		86	1.00	
Exclusive waterpipe	30,957	361	1.63 (1.32, 2.00)	0.000	126	1.67 (1.18, 2.38)	0.004
Mixed waterpipe cigarette smoking	27,893	275	1.75 (1.40, 2.19)	0.000	73	1.18 (0.80, 1.75)	0.401

Adjusted for age groups (10–19, 20–29, 30–39, 40–49, 50–59, 60–69, 70–79, 80 +); Education level (< 6 years, 7 + years, unknown); Available fridge at home (yes/no, unknown); BMI (kg/m^2^, < 18.5, 18.5- < 23, 23 + , unknown); Alcohol consumption (yes/no, unknown); Family history of cancer; Age at started smoking; Cumulative number of smokes lifetime; Years of quit smoking; Total energy intake (Kcal/day, quintiles); Protein intake (g/day, quintiles); Lipid intake (g/day, quintiles); Carbohydrate intake (g/day, quintiles). # adjusted for “&” and additional health history of hypertension.

Continuing to take the ever-cigarette smoking group as the reference group, dose–response analysis for three levels of gradual increase in a smoking duration shorter than 11 years, 11–20 years, and over 20 years, the risk of death increased steadily. The risk of death increased statistically significantly in the group of waterpipe smoking over 20 years for overall mortality HR (95%CI): 1.82 (1.45, 2.29), and all cancers HR (95%CI): 1.91 (1.27, 2.88), Table 4**.**

**Table 4:** Compared to exclusive cigarette smokers, the risk of death due to all causes and cancer among men waterpipe tobacco smokers, a dose-respond by smoking years.

			All causes			All cancers	
Smoking years	person-year	Cases	Multivariable adjusted HR (95%CI) #	*P *for trend	Cases	Multivariable adjusted HR (95%CI) &	*P *for trend
Ever cigarette smoking	36,227	249	1.00		86	1.00	
Less than 11 years	23,466	129	1.36 (1.02, 1.80)		38	0.84 (0.52, 1.37)	
11–20 years	17,092	165	1.77 (1.39, 2.25)		64	1.69 (1.14, 2.50)	
Over 20 years	14,673	312	1.82 (1.45, 2.29)	0.000	92	1.91 (1.27, 2.88)	0.001

Adjusted for age groups (10–19, 20–29, 30–39, 40–49, 50–59, 60–69, 70–79, 80 +); Education level (< 6 years, 7 + years, unknown); Available fridge at home (yes/no, unknown); BMI (kg/m^2^, < 18.5, 18.5- < 23, 23 + , unknown); Alcohol consumption (yes/no, unknown); Family history of cancer; Age at started smoking; Cumulative number of smokes lifetime; Years of quit smoking; Total energy intake (Kcal/day, quintiles); Protein intake (g/day, quintiles); Lipid intake (g/day, quintiles); Carbohydrate intake (g/day, quintiles). # adjusted for “&” and additional health history of hypertension.

After quitting waterpipe smoking, the risk of death decreased steadily. The risk of death was reduced by 41% for overall mortality HR (95%CI): 0.59 (0.39, 0.89), and 74% for death from cancers HR (95%CI): 0.26 (0.08, 0.83) among smokers who have stopped smoking successfully for ten years or longer, Table 5.

**Table 5:** Hazards of deaths due to all causes and cancers among exclusive waterpipe smokers after cessation in men.

			All causes			All cancers	
Time to stop smoking	person-year	Cases	Multivariable adjusted HR (95%CI) #	*P *for trend	Cases	Multivariable adjusted HR (95%CI) &	*P *for trend
Less than four years	2853	50	1.00		12	1.00	
4–7 years	2060	36	0.85 (0.55, 1.32)		9	1.07 (0.44, 2.58)	
8–10 years	2340	42	0.76 (0.50, 1.15)		11	0.96 (0.41, 2.21)	
Over ten years	2289	46	0.59 (0.39, 0.89)	0.011	4	0.26 (0.08, 0.83)	0.033

Adjusted for age groups (10–19, 20–29, 30–39, 40–49, 50–59, 60–69, 70–79, 80 +); Education level (< 6 years, 7 + years, unknown); Available fridge at home (yes/no, unknown); BMI (kg/m^2^, < 18.5, 18.5- < 23, 23 + , unknown); Alcohol consumption (yes/no, unknown); Family history of cancer; Total energy intake (Kcal/day, quintiles); Protein intake (g/day, quintiles); Lipid intake (g/day, quintiles); Carbohydrate intake (g/day, quintiles). # adjusted for “&” and additional health history of hypertension.

**Figure 2:**
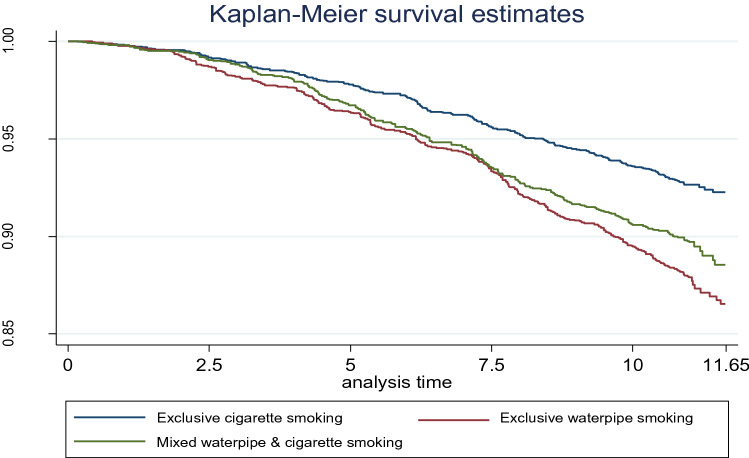
Kaplan–Meier survival estimates by types of tobacco smoking of exclusive cigarette smoking (higher line), mixed waterpipe & cigarette smoking (Medium line), and exclusive waterpipe smoking (Lower line).

Life expectancy was shortened by 6.1 years, 4.6 years, and 2.2 years for the groups of exclusive waterpipe smokers, both waterpipe and cigarette smokers, and exclusive cigarette smokers, respectively, compared with non-smokers. The survival curve was lower in the group of exclusive waterpipe smokers than in the group of both waterpipe and cigarette smokers and much lower when compared with the group of exclusive cigarette smokers, Fig. 2.

## Discussions

The current prospective cohort study has yielded many new results. First, waterpipe smokers are more likely to become addicted than cigarette smokers. This new finding is consistent with previous laboratory results of higher concentrations of chemicals in waterpipe smoke than in cigarettes in terms of nicotine, carbon monoxide, carbonylic compounds, heavy metals, tar, and polyaromatic hydrocarbons^3^. The prevalence of exclusive waterpipe smokers was close to that of exclusive cigarette smokers in this study population of men.

Second, we found a statistically increased risk of death from all causes and cancers due to exclusive waterpipe smoking compared with exclusive cigarette smoking. This superior level of danger was even more evident in a dose–response analysis, with waterpipe smoking significantly increasing the overall risk of death by 82% and the risk of dying from all types of cancer by 91% among waterpipe smokers who have smoked for over 20 years. The loss of life expectancy of exclusive waterpipe smokers is over six years compared to the never-smokers group. These new findings provide clear and convincing evidence of the overwhelming dangers of waterpipe smoking and its impact on potential premature death and loss of many years of life. This new finding has consistent with the previous results on the role of waterpipe smoking in the development of gastric cancer in Viet Nam^8,9,12^. The findings also supported the last observation in the United States and European Countries^6,7^.

In addition to the two critical new findings above, the present study confirms an increased risk of cancer death from tobacco and waterpipe tobacco. These findings have consistent with previous studies^3,6,7,12–15^. Like cigarette smoking, after cessation of waterpipe tobacco exposure, the risk of death from all causes and all types of cancers decreased rapidly and statistically, consistent with another study^6^.

The advantage of this study is the design of a prospective cohort study for the population participating in the study with a total number of over 20,000 men aged ten and older. Advanced adjustments for the diet of macro-nutrients and other demographic indicators and the health history of each study participant were well managed in this study. The follow-up period for smoking-related illness and death is nearly 12 years to measure the association between waterpipe smoking and overall mortality and cancers. For men, the total number of deaths was 1,413, of which 882 were in smokers, allowing analyses with a sample force strong enough for the new findings in this study.

Some limitations of this study include that the determination of smoking status was confirmed only once by a baseline survey. Incidence data as the outcome is not available in the study. The other limitation is no available data analysis on female smoking due to the small sample size.

In conclusion, this study found a new novel danger and hazard of exclusive waterpipe tobacco smoking. The new findings of this study serve as scientific evidence for developing strategies, policies, and budget allocations for control to reduce tobacco and waterpipe tobacco production, promote smoking cessation, prevent new smokers, increase screening for early detection of diseases related to tobacco smoking, especially cancer in ever waterpipe smokers, to reduce the number of new incidence cases and deaths related to tobacco smoking in Vietnam and other countries around the world.

## Data Availability

Data is available from the corresponding author on request.
